# COVID-19–Associated Multisystem Inflammatory Syndrome in Children — United States, March–July 2020

**DOI:** 10.15585/mmwr.mm6932e2

**Published:** 2020-08-14

**Authors:** Shana Godfred-Cato, Bobbi Bryant, Jessica Leung, Matthew E. Oster, Laura Conklin, Joseph Abrams, Katherine Roguski, Bailey Wallace, Emily Prezzato, Emilia H. Koumans, Ellen H. Lee, Anita Geevarughese, Maura K. Lash, Kathleen H. Reilly, Wendy P. Pulver, Deepam Thomas, Kenneth A. Feder, Katherine K. Hsu, Nottasorn Plipat, Gillian Richardson, Heather Reid, Sarah Lim, Ann Schmitz, Timmy Pierce, Susan Hrapcak, Deblina Datta, Sapna Bamrah Morris, Kevin Clarke, Ermias Belay, California MIS-C Response Team

**Affiliations:** ^1^CDC COVID-19 Response Team; ^2^Oak Ridge Institute for Science and Education; ^3^New York City Department of Health and Mental Hygiene; ^4^New York State Department of Health; ^5^New Jersey Department of Health; ^6^Epidemic Intelligence Service, Prevention and Health Promotion Administration, Maryland Department of Health; ^7^Massachusetts Department of Public Health; ^8^Pennsylvania Department of Health; ^9^Louisiana Department of Health; ^10^Illinois Department of Public Health; ^11^Minnesota Department of Health; ^12^Florida Department of Health; ^13^Career Epidemiology Field Officer Program, CDC.

In April 2020, during the peak of the coronavirus disease 2019 (COVID-19) pandemic in Europe, a cluster of children with hyperinflammatory shock with features similar to Kawasaki disease and toxic shock syndrome was reported in England* (*1*). The patients’ signs and symptoms were temporally associated with COVID-19 but presumed to have developed 2–4 weeks after acute COVID-19; all children had serologic evidence of infection with SARS-CoV-2, the virus that causes COVID-19 (*1*). The clinical signs and symptoms present in this first cluster included fever, rash, conjunctivitis, peripheral edema, gastrointestinal symptoms, shock, and elevated markers of inflammation and cardiac damage (*1*). On May 14, 2020, CDC published an online Health Advisory that summarized the manifestations of reported multisystem inflammatory syndrome in children (MIS-C), outlined a case definition,^†^ and asked clinicians to report suspected U.S. cases to local and state health departments. As of July 29, a total of 570 U.S. MIS-C patients who met the case definition had been reported to CDC. A total of 203 (35.6%) of the patients had a clinical course consistent with previously published MIS-C reports, characterized predominantly by shock, cardiac dysfunction, abdominal pain, and markedly elevated inflammatory markers, and almost all had positive SARS-CoV-2 test results. The remaining 367 (64.4%) of MIS-C patients had manifestations that appeared to overlap with acute COVID-19 (*2*–*4*), had a less severe clinical course, or had features of Kawasaki disease.^§^ Median duration of hospitalization was 6 days; 364 patients (63.9%) required care in an intensive care unit (ICU), and 10 patients (1.8%) died. As the COVID-19 pandemic continues to expand in many jurisdictions, clinicians should be aware of the signs and symptoms of MIS-C and report suspected cases to their state or local health departments; analysis of reported cases can enhance understanding of MIS-C and improve characterization of the illness for early detection and treatment.

Local and state health departments reported suspected MIS-C patients to CDC using CDC’s MIS-C case report form, which included information on patient demographics, clinical findings, and laboratory test results. Patients who met the MIS-C case definition and were reported to CDC as of July 29, 2020, were included in the analysis. Latent class analysis (LCA), a statistical modeling technique that can divide cases into groups by underlying similarities, was used to identify and describe differing manifestations in patients who met the MIS-C case definition. The indicator variables used in the LCA were the presence or absence of SARS-CoV-2–positive test results by reverse transcription–polymerase chain reaction (RT-PCR) or serology, shock, pneumonia, and involvement of organ systems (i.e., cardiovascular, dermatologic, gastrointestinal, hematologic, neurologic, renal, or respiratory). Three-class LCA was conducted using the R software package “poLCA” with 100 iterations to identify the optimal classification scheme (*5*). Clinical and demographic variables were reported for patients by LCA class. Chi-squared or Fisher’s exact tests were used to compare proportions of categorical variables; numeric variables, with medians and interquartile ranges, were compared using the Kruskal-Wallis rank sum test.

As of July 29, 2020, a total of 570 MIS-C patients with onset dates from March 2 to July 18, 2020, had been reported from 40 state health departments, the District of Columbia, and New York City (Figure). The median patient age was 8 years (range = 2 weeks–20 years); 55.4% were male, 40.5% were Hispanic or Latino (Hispanic), 33.1% were non-Hispanic black (black), and 13.2% non-Hispanic white (white) (Table 1). Obesity was the most commonly reported underlying medical condition, occurring in 30.5% of Hispanic, 27.5% of black, and 6.6% of white MIS-C patients.

**FIGURE:**
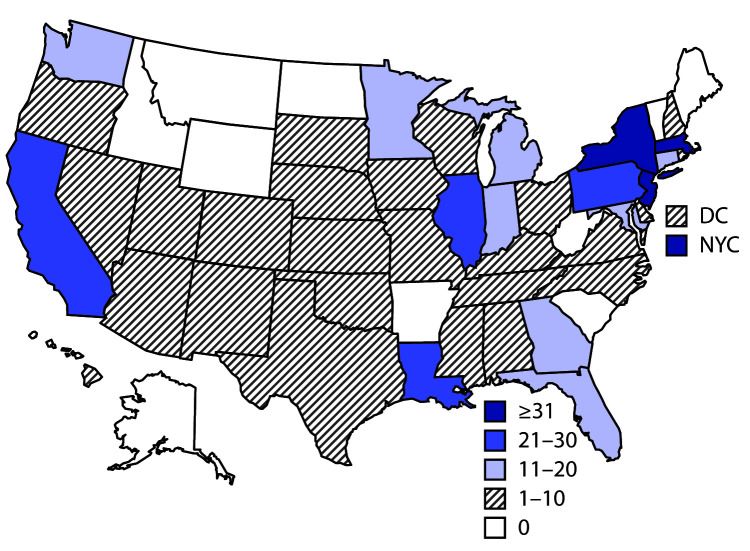
Geographic distribution of 570 reported cases of multisystem inflammatory syndrome in children — United States, March–July 2020 **Abbreviations:** DC = District of Columbia; NYC = New York City.

**TABLE 1:** Characteristics of patients (N = 570) reported with multisystem inflammatory syndrome in children (MIS–C) — United States, March–July 2020

Characteristic	No. (%)				p value
	Total (N = 570)	Latent class analysis group*			
		Class 1 (n = 203)	Class 2 (n = 169)	Class 3 (n = 198)	
**Sex**					
Female	254 (44.6%)	87 (42.9%)	81 (47.9%)	86 (43.4%)	0.57
Male	316 (55.4%)	116 (57.1%)	88 (52.1%)	112 (56.6%)	
Age (yrs), median (IQR)	8 (4–12)	9 (6–13)	10 (5–15)	6 (3–10)	<0.01
**Race/Ethnicity**					
Hispanic	187 (40.5%)	62 (36.9%)	62 (46.6%)	63 (39.1%)	0.03
Black, non–Hispanic	153 (33.1%)	66 (39.3%)	39 (29.3%)	48 (29.8%)	
White, non–Hispanic	61 (13.2%)	22 (13.1%)	15 (11.3%)	24 (14.9%)	
Other	26 (5.6%)	8 (4.8%)	6 (4.5%)	12 (7.5%)	
Multiple	18 (3.9%)	9 (5.4%)	5 (3.8%)	4 (2.5%)	
Asian	13 (2.8%)	1 (0.6%)	3 (2.3%)	9 (5.6%)	
American Indian/Alaskan Native	3 (0.6%)	0 (0.0%)	3 (2.3%)	0 (0.0%)	
Native Hawaiian/Pacific Islander	1 (0.2%)	0 (0.0%)	0 (0.0%)	1 (0.6%)	
Unknown	108 (─)	35 (─)	36 (─)	37 (─)	
**Outcome**					
Died	10 (1.8%)	1 (0.5%)	9 (5.3%)	0 (0.0%)	<0.01
**Days in hospital, median (IQR)**	6 (4–9)	8 (6–11)	6 (4–10)	5 (4–8)	<0.01
1	16 (3.2%)	3 (1.8%)	3 (2.0%)	10 (5.4%)	<0.01
2–7	304 (60.2%)	86 (50.3%)	87 (58.8%)	131 (70.4%)	
8–14	149 (29.5%)	66 (38.6%)	41 (27.7%)	42 (22.6%)	
≥15	36 (7.1%)	16 (9.4%)	17 (11.5%)	3 (1.6%)	
Missing	65 (─)	32 (─)	21 (─)	12 (─)	
**ICU admission**	364 (63.9%)	171 (84.2%)	105 (62.1%)	88 (44.4%)	<0.01
**Days in ICU, median (IQR)**	5 (3–7)	5 (4–7)	6 (3–9)	3 (2–5)	<0.01
**Underlying medical conditions**					<0.01
Obesity	146 (25.6%)	60 (29.6%)	49 (29.0%)	37 (18.7%)	0.02
Chronic lung disease	48 (8.4%)	18 (8.9%)	17 (10.1%)	13 (6.6%)	0.46
**Clinical characteristic**					
**No. of organ systems involved**					
2–3	80 (14.0%)	6 (3.0%)	24 (14.2%)	50 (25.3%)	<0.01
4–5	351 (61.6%)	98 (48.3%)	113 (66.9%)	140 (70.7%)	
≥6	139 (24.4%)	99 (48.8%)	31 (18.3%)	9 (4.5%)	
**Days with fever, median (IQR)**	5 (3–6)	5 (3–6)	5 (3–6)	5 (3–6)	0.81
**Kawasaki disease^†^**	28 (4.9)	10 (4.9)	5 (3.0)	13 (6.6)	0.30
**Organ system involvement**					
**Gastrointestinal**	518 (90.9%)	198 (97.5%)	146 (86.4%)	174 (87.9%)	<0.01
Abdominal pain	353 (61.9%)	163 (80.3%)	83 (49.1%)	107 (54.0%)	<0.01
Vomiting	352 (61.8%)	145 (71.4%)	95 (56.2%)	112 (56.6%)	<0.01
Diarrhea	303 (53.2%)	124 (61.1%)	79 (46.7%)	100 (50.5%)	0.01
**Cardiovascular**	493 (86.5%)	203 (100.0%)	143 (84.6%)	147 (74.2%)	<0.01
Shock	202 (35.4%)	154 (75.9%)	48 (28.4%)	0 (0.0%)	<0.01
Elevated troponin	176 (30.9%)	93 (45.8%)	43 (25.4%)	40 (20.2%)	<0.01
Elevated BNP or NT–proBNP	246 (43.2%)	105 (51.7%)	77 (45.6%)	64 (32.3%)	<0.01
Congestive heart failure	40 (7.0%)	21 (10.3%)	14 (8.3%)	5 (2.5%)	0.02
Cardiac dysfunction^§^	207 (40.6%)	105 (55.3%)	64 (46.0%)	38 (21.0%)	<0.01
Myocarditis	130 (22.8%)	62 (30.5%)	36 (21.3%)	32 (16.2%)	0.01
Coronary artery dilatation or aneurysm^§^	95 (18.6%)	40 (21.1%)	22 (15.8%)	33 (18.2%)	0.49
Hypotension	282 (49.5%)	162 (79.8%)	75 (44.4%)	45 (22.7%)	<0.01
Pericardial effusion^§^	122 (23.9%)	55 (28.9%)	32 (23.0%)	35 (19.3%)	0.01
Mitral regurgitation^§^	130 (25.5%)	68 (35.8%)	30 (21.6%)	32 (17.7%)	<0.01
**Dermatologic and mucocutaneous**	404 (70.9%)	156 (76.8%)	87 (51.5%)	161 (81.3%)	<0.01
Rash	315 (55.3%)	121 (59.6%)	70 (41.4%)	124 (62.6%)	<0.01
Mucocutaneous lesions	201 (35.3%)	70 (34.5%)	42 (24.9%)	89 (44.9%)	<0.01
Conjunctival injection	276 (48.4%)	118 (58.1%)	54 (32.0%)	104 (52.5%)	<0.01
**Hematologic**	421 (73.9%)	161 (79.3%)	130 (76.9%)	130 (65.7%)	<0.01
Elevated D–dimer	344 (60.4%)	136 (67.0%)	104 (61.5%)	104 (52.5%)	0.01
Thrombocytopenia^¶^	176 (30.9%)	84 (41.4%)	45 (26.6%)	47 (23.7%)	<0.01
Lymphopenia^¶^	202 (35.4%)	82 (40.4%)	60 (35.5%)	60 (30.3%)	0.11
Respiratory**	359 (63.0%)	155 (76.4%)	129 (76.3%)	75 (37.9%)	<0.01
Cough	163 (28.6%)	51 (25.1%)	67 (39.6%)	45 (22.7%)	<0.01
Shortness of breath	149 (26.1%)	66 (32.5%)	59 (34.9%)	24 (12.1%)	<0.01
Chest pain or tightness	66 (11.6%)	33 (16.3%)	24 (14.2%)	9 (4.5%)	0.01
Pneumonia^††^	110 (19.3%)	47 (23.2%)	62 (36.7%)	1 (0.5%)	<0.01
ARDS	34 (6.0%)	14 (6.9%)	17 (10.1%)	3 (1.5%)	<0.01
Pleural effusion^§§^	86 (15.8%)	49 (24.7%)	29 (18.4%)	8 (4.2%)	<0.01
**Neurologic**	218 (38.2%)	107 (52.7%)	70 (41.4%)	41 (20.7%)	<0.01
Headache	186 (32.6%)	90 (44.3%)	63 (37.3%)	33 (16.7%)	<0.01
**Renal**	105 (18.4%)	77 (37.9%)	28 (16.6%)	0 (0.0%)	<0.01
Acute kidney injury	105 (18.4%)	77 (37.9%)	28 (16.6%)	0 (0.0%)	<0.01
**Other**					
Periorbital edema	27 (4.7%)	13 (6.4%)	5 (3.0%)	9 (4.5%)	0.32
Cervical lymphadenopathy >1.5 cm diameter	76 (13.3%)	28 (13.8%)	18 (10.7%)	30 (15.2%)	0.43
**SARS COV–2 testing**					
Any laboratory test done	565 (99.1%)	200 (98.5%)	169 (100.0%)	196 (99.0%)	0.39
Any positive laboratory test^¶¶^ (% among tested)	565 (100.0%)	200 (100.0%)	169 (100.0%)	196 (100.0%)	NA
PCR positive/Serology negative, not done, or missing***	147 (25.8%)	1 (0.5%)	142 (84.0%)	4 (2.0%)	<0.01
Serology positive/PCR negative^†††^	263 (46.1%)	138 (68.0%)	0 (0.0%)	125 (63.1%)	<0.01
PCR positive/Serology positive	155 (27.2%)	61 (30.0%)	27 (16.0%)	67 (33.8%)	<0.01
Epidemiologic link only, with no testing	5 (0.9%)	3 (1.5%)	0 (0.0%)	2 (1.0%)	<0.01
**Treatment** ^§§§^					
IVIG^¶¶¶^	424 (80.5%)	174 (87.9%)	96 (62.7%)	154 (87.5%)	<0.01
Steroids	331 (62.8%)	145 (73.2%)	80 (52.3%)	106 (60.2%)	<0.01
Antiplatelet medication	309 (58.6%)	113 (57.1%)	69 (45.1%)	127 (72.2%)	<0.01
Anticoagulation medication	233 (44.2%)	92 (46.5%)	76 (49.7%)	65 (36.9%)	0.03
Vasoactive medications	221 (41.9%)	129 (65.2%)	64 (41.8%)	28 (15.9%)	<0.01
Respiratory support, any	201 (38.1%)	104 (52.5%)	79 (51.6%)	18 (10.2%)	<0.01
Intubation and mechanical ventilation	69 (13.1%)	37 (18.7%)	30 (19.6%)	2 (1.1%)	<0.01
Immune modulators	119 (22.6%)	52 (26.3%)	34 (22.2%)	33 (18.8%)	0.18
Dialysis	2 (0.4%)	0 (0.0%)	2 (1.3%)	0 (0.0%)	0.08

**Abbreviations:** ARDS = acute respiratory distress syndrome; BNP = brain natriuretic peptide; ICU = intensive care unit; IQR = interquartile range; IVIG = intravenous immune globulin; NT-proBNP = N-terminal pro b-type natriuretic peptide; PCR = polymerase chain reaction.

* Latent class analysis (LCA) is a statistical modeling technique in which observations can be classified into latent classes based on their underlying similarities. Variables that are associated with MIS-C clinical manifestation were selected as indicator variables and included in the LCA model.

^†^ Patient had fever, rash, conjunctival injection, cervical lymphadenopathy >1.5 cm diameter, and mucocutaneous lesions.

^§^ Percentages calculated among 510 persons with an echocardiogram performed.

^¶^ Thrombocytopenia was defined as a platelet count of less than 150 x 10^3^ per *μ*l or if thrombocytopenia was checked on the case-report form. Lymphopenia was defined as a lymphocyte count of <4,500 cells per *μ*l for infants aged <8 months, or less than 1,500 cells per ml for persons aged ≥8 months.

**Among 359 with respiratory organ system involvement, 324 (90%) also had cardiovascular system involvement.

^††^ Information about pneumonia was collected on the case report form under signs and symptoms, complications, or chest imaging.

^§§^ Percentages calculated among 545 persons with either an echocardiogram or chest imaging performed.

^¶¶^ Eight cases had a positive SARS CoV–2 antigen test result, among whom three were also positive by both PCR and serology, one was positive by PCR alone, and one was positive by serology alone.

*** Among 147 cases with a positive PCR result without a positive serologic test result, 10 had a negative serologic test, and the remaining had unknown serologic testing.

^†††^ Among 263 cases with positive serologic test result without a positive PCR result, 254 had a negative PCR result, and the remaining had unknown PCR testing.

^§§§^ Percentages calculated among 527 persons who received treatment.

^¶¶¶^ 73 received a second dose of IVIG.

Overall, the illness in 490 (86.0%) patients involved four or more organ systems. Approximately two thirds did not have preexisting underlying medical conditions before MIS-C onset. The most common signs and symptoms reported during illness course were abdominal pain (61.9%), vomiting (61.8%), skin rash (55.3%), diarrhea (53.2%), hypotension (49.5%), and conjunctival injection (48.4%). Most patients had gastrointestinal (90.9%), cardiovascular (86.5%), or dermatologic or mucocutaneous (70.9%) involvement. Substantial numbers of MIS-C patients had severe complications, including cardiac dysfunction (40.6%), shock (35.4%), myocarditis (22.8%), coronary artery dilatation or aneurysm (18.6%), and acute kidney injury (18.4%). The majority of patients (63.9%) were admitted to an ICU. The median length of ICU stay was 5 days (interquartile range = 3–7 days).

Of the 565 (99.1%) patients who underwent SARS-CoV-2 testing, all had a positive test result by RT-PCR or serology; 46.1% had only serologic evidence of infection and 25.8% had only positive RT-PCR test results. Five patients (0.9%) did not have testing performed but had an epidemiologic link as indicated in the MIS-C case definition.

Among all 570 patients, 527 (92.5%) were treated, including 424 (80.5%) who received intravenous immunoglobulin (IVIG), 331 (62.8%) who received steroids, 309 (58.6%) who received antiplatelet medication, 233 (44.2%) who received anticoagulation medication, and 221 (41.9%) who were treated with vasoactive medication. Ten (1.8%) patients were reported to have died (Table 1).

LCA identified three classes of patients, each of which had significantly different illness manifestations related to some of the key indicator variables. Class 1 represented 203 (35.6%) patients who had the highest number of involved organ systems. Within this group, 99 (48.8%) had involvement of six or more organ systems; those most commonly affected were cardiovascular (100.0%) and gastrointestinal (97.5%). Compared with the other classes, patients in class 1 had significantly higher prevalences of abdominal pain, shock, myocarditis, lymphopenia, markedly elevated C-reactive protein (produced in the liver in response to inflammation), ferritin (an acute-phase reactant), troponin (a protein whose presence in the blood indicates possible cardiac damage), brain natriuretic peptide (BNP), or proBNP (indicative of heart failure) (p<0.01) (Tables 1 and 2). Almost all class 1 patients (98.0%) had positive SARS-CoV-2 serology test results with or without positive SARS-CoV-2 RT-PCR test results. These cases closely resembled MIS-C without overlap with acute COVID-19 or Kawasaki disease.

**TABLE 2:** Reported serum laboratory values for multisystem inflammatory syndrome in children (MIS-C) cases (N = 570), by latent class analysis (LCA) group* — United States, March–July 2020

	LCA class 1			LCA class 2			LCA class 3			p-value
Laboratory test	No.	Median	IQR	No.	Median	IQR	No.	Median	IQR	
Fibrinogen, peak (mg/dL)	151	557	(449–713)	87	566	(430–662)	105	546	(426–681)	0.67
D-dimer, peak (mg/L)	158	3.0	(1.6–4.9)	106	2.6	(1.2–5.1)	128	1.7	(0.8–3.2)	<0.01
Troponin, peak (ng/mL)	162	0.09	(0.02–0.48)	109	0.05	(0.01–0.30)	130	0.01	(0.01–0.08)	<0.01
BNP, peak (pg/mL)	53	1,321	(414–2,528)	30	198	(76–927)	25	182	(30–616)	<0.01
proBNP, peak (ng/L)	103	4,700	(1,261–13,646)	68	1,503	(247–6,846)	92	507	(176–2,153)	<0.01
CRP, peak (mg/L)	166	21	(14–29)	122	16	(9–25)	144	14	(6–23)	<0.01
Ferritin, peak (ng/mL)	159	610	(347–1,139)	108	422	(207–825)	132	242	(116–466)	<0.01
IL-6, peak (pg/mL)	54	65	(24–258)	27	41	(21–131)	29	69	(7–118)	0.24
Platelets, nadir (10^3^ cells/μl)	115	131	(102–203)	76	172	(103–245)	68	150	(113–237)	0.15
Lymphocytes, nadir (cells/μl)	72	695	(400–1,093)	49	1,200	(790–2,025)	42	1,420	(723–2,250)	<0.01

**Abbreviations:** BNP = brain natriuretic peptide; CRP = C-reactive protein; IL-6 = Interleukin-6; IQR = interquartile range.

* Latent class analysis (LCA) is a statistical modeling technique in which observations can be classified into latent classes based on their underlying similarities. Variables that are associated with MIS-C clinical manifestation were selected as indicator variables and included in the LCA model.

Class 2 included 169 (29.6%) patients; among those in this group, 129 (76.3%) had respiratory system involvement. These patients were significantly more likely to have cough, shortness of breath, pneumonia, and acute respiratory distress syndrome (ARDS), indicating that their illnesses might have been primarily acute COVID-19 or a combination of acute COVID-19 and MIS-C. The rate of SARS-CoV-2 RT-PCR positivity (without seropositivity) in this group (84.0%) was significantly higher than that for class 1 (0.5%) or class 3 (2.0%) patients (p<0.01). The case fatality rate among class 2 patients was the highest (5.3%) among all three classes (p<0.01).

Class 3 included 198 (34.7%) patients; the median age of children in this group (6 years) was younger than that of the class 1 patients (9 years) or class 2 patients (10 years) (p<0.01) (Table 1). Class 3 patients also had the highest prevalence of rash (62.6%), and mucocutaneous lesions (44.9%). Although not statistically significant (p = 0.49), the prevalence of coronary artery aneurysm and dilatations (18.2%) was higher than that in class 2 patients (15.8%), but lower than that in class 1 patients (21.1%). Class 3 patients more commonly met criteria for complete Kawasaki disease (6.6%) compared with class 1 (4.9%) and class 2 (3.0%) patients (p = 0.30), and had the lowest prevalence of underlying medical conditions, organ system involvement, complications (e.g., shock, myocarditis), and markers of inflammation and cardiac damage. Among class 3 patients, 63.1% had positive SARS-CoV-2 serology only and 33.8% had both serologic confirmation and positive RT-PCR results.

## Discussion

Initial reports of MIS-C patients described varied clinical signs and symptoms at initial evaluation, but most cases included features of shock, cardiac dysfunction, gastrointestinal symptoms, significantly elevated markers of inflammation and cardiac damage, and positive test results for SARS-CoV-2 by serology (*3*,*6*–*8*). Because the case definition is nonspecific and confirmatory laboratory testing does not exist, it might be difficult to distinguish MIS-C from other conditions with overlapping clinical manifestations such as severe acute COVID-19 and Kawasaki disease (*9*). Latent class analysis is particularly well-suited to describe differing manifestations of a novel clinical syndrome. It divides patients into groups that might have been previously unrecognized, based on shared characteristics, allowing for an unbiased determination of disease manifestations. Patients identified in class 1 had little overlap with acute COVID-19 or Kawasaki disease, whereas patients in class 2 had clinical and laboratory manifestations that overlapped with acute COVID-19. This overlap might result from the development of MIS-C soon after symptomatic acute COVID-19 illness. However, the presence of isolated severe acute COVID-19 illness cannot be ruled out in some of these patients. Patients in class 3 generally seemed to have less severe MIS-C illness and clinical manifestations that overlapped with Kawasaki disease, and distinguishing class 3 patients from those with true Kawasaki disease could be difficult (*4*). As the COVID-19 pandemic spreads, and more children are exposed to SARS-CoV-2 with subsequent seroconversion, patients with Kawasaki disease might be misidentified as MIS-C because of an incidental finding of antibodies to SARS-CoV-2.

Overall, the age distribution of the patients in this analysis is similar to that described elsewhere, but there are differences in the clinical manifestations and laboratory findings, perhaps due to differences in inclusion criteria (*6*,*7*). Increases in COVID-19 incidence might result in increased occurrence of MIS-C which might not be apparent immediately because of the 2–4-week delay in the development of MIS-C after acute SARS-CoV-2 infection (*8*). The proportion of Hispanic, black, and white MIS-C patients with obesity is slightly higher than that reported in the general pediatric population.^¶^ Hispanic and black patients accounted for the largest proportion (73.6%) of reported MIS-C patients. Acute COVID-19 has been reported to disproportionately affect Hispanics and blacks (*10*). Long-standing inequities in the social determinants of health, such as housing, economic instability, insurance status, and work circumstances of patients and their family members have systematically placed social, racial, and ethnic minority populations at higher risk for COVID-19 and more severe illness, possibly including MIS-C.**

The findings in this report are subject to at least four limitations. First, there is a possibility of case identification and reporting bias, including variability in diagnosis, testing, and management of patients by different jurisdictions. Second, inconsistency in completion of case report forms, with some patients still hospitalized at the time of reporting, might have affected data completeness (e.g., race and ethnicity were not reported for 18.9% of cases). Third, access to SARS-CoV-2 testing at the time of onset might have varied by regions, hospitals, and time. Finally, CDC’s case definition was broad, with the intention of being more inclusive, which might have led to the unintentional inclusion of patients whose illnesses overlapped with acute COVID-19 and Kawasaki disease.

As the COVID-19 pandemic continues, with the number of cases increasing in many jurisdictions, health care providers should continue to monitor patients to identify children with a hyperinflammatory syndrome with shock and cardiac involvement. Suspected MIS-C patients should be reported to local and state health departments. Distinguishing patients with MIS-C from those with acute COVID-19 and other hyperinflammatory conditions is critical for early diagnosis and appropriate management. It is also critical for monitoring potential adverse events of a COVID-19 vaccine when one becomes widely available. Studies to define the clinical and laboratory characteristics of MIS-C should continue, including identification of parameters that will help distinguish the illness from other similar conditions.

**Summary**

**What is already known about this topic?**

Multisystem inflammatory syndrome in children (MIS-C) is a rare but severe condition that has been reported approximately 2–4 weeks after the onset of COVID-19 in children and adolescents.

**What is added by this report?**

Most cases of MIS-C have features of shock, with cardiac involvement, gastrointestinal symptoms, and significantly elevated markers of inflammation, with positive laboratory test results for SARS-CoV-2. Of the 565 patients who underwent SARS-CoV-2 testing, all had a positive test result by RT-PCR or serology.

**What are the implications for public health practice?**

Distinguishing MIS-C from other severe infectious or inflammatory conditions poses a challenge to clinicians caring for children and adolescents. As the COVID-19 pandemic continues to expand in many jurisdictions, health care provider awareness of MIS-C will facilitate early recognition, early diagnosis, and prompt treatment.

## Footnotes

*
https://www.rcpch.ac.uk/sites/default/files/2020-05/COVID-19-Paediatric-multisystem-%20inflammatory%20syndrome-20200501.pdf .

† The MIS-C case definition included a patient aged <21 years with fever, laboratory evidence of inflammation, and evidence of clinically severe illness requiring hospitalization, with multisystem organ involvement (cardiovascular, dermatologic, gastrointestinal, hematologic, neurologic, renal, or respiratory) who tested positive for SARS-CoV-2 or had exposure to COVID-19. https://www.cdc.gov/mis-c/hcp/.

§ Kawasaki disease is an acute febrile illness of unknown cause, primarily affecting children, and associated with fever, rash, conjunctivitis, redness in the mouth, cracked lips, and swollen lymph nodes, feet, and hands.

¶
https://www.cdc.gov/obesity/data/childhood.html .

** https://www.cdc.gov/coronavirus/2019-ncov/community/health-equity/race-ethnicity.html?CDC_AA_refVal=https%3A%2F%2Fwww.cdc.gov%2Fcoronavirus%2F2019-ncov%2Fneed-extra-precautions%2Fracial-ethnic-minorities.html.
