# *Erratum:* Vol. 69, No. 32

**DOI:** 10.15585/mmwr.mm6935a6

**Published:** 2020-09-04

In the report “COVID-19–Associated Multisystem Inflammatory Syndrome in Children — United States, March–July 2020,” on page 1078, in Table 2, under “Laboratory test,” the test listed in the sixth row should have read “CRP, peak (mg/**dL**).”

